# Exploring Children's Knowledge of Healthy Eating, Digital Media Use, and Caregivers’ Perspectives to Inform Design and Contextual Considerations for Game-Based Interventions in Schools for Low-Income Families in Lima, Peru: Survey Study

**DOI:** 10.2196/49168

**Published:** 2024-05-14

**Authors:** Bladimir Morales-Cahuancama, Nervo Verdezoto, Elena Gonzales-Achuy, Cinthia Quispe-Gala, William Bautista-Olortegui, Paul Hinojosa-Mamani, Juan Pablo Aparco

**Affiliations:** 1 Centro Nacional de Alimentación, Nutrición y Vida Saludable Instituto Nacional de Salud Lima Peru; 2 Programa Académico de Nutrición y Dietética Facultad de ciencias de la salud Universidad Peruana de Ciencias Aplicadas Lima Peru; 3 School of Computer Science and Informatics Cardiff University Cardiff United Kingdom; 4 Escuela Profesional de Nutrición Facultad de Medicina Universidad Nacional Mayor de San Marcos Lima Peru

**Keywords:** child, children, schoolchildren, youth, student, students, adolescent, schoolchildren, formative research, digital media, digital games, serious game, serious games, nutrition, obesity, obese, overweight, mHealth, caregivers’ perspectives, perspective, perspectives, diet, healthy eating

## Abstract

**Background:**

The prevalence of overweight and obesity in schoolchildren is increasing in Peru. Given the increased use of digital media, there is potential to develop effective digital health interventions to promote healthy eating practices at schools. This study investigates the needs of schoolchildren in relation to healthy eating and the potential role of digital media to inform the design of game-based nutritional interventions.

**Objective:**

This study aims to explore schoolchildren’s knowledge about healthy eating and use of and preferences for digital media to inform the future development of a serious game to promote healthy eating.

**Methods:**

A survey was conducted in 17 schools in metropolitan Lima, Peru. The information was collected virtually with specific questions for the schoolchild and their caregiver during October 2021 and November 2021 and following the COVID-19 public health restrictions. Questions on nutritional knowledge and preferences for and use of digital media were included. In the descriptive analysis, the percentages of the variables of interest were calculated.

**Results:**

We received 3937 validated responses from caregivers and schoolchildren. The schoolchildren were aged between 8 years and 15 years (2030/3937, 55.8% girls). Of the caregivers, 83% (3267/3937) were mothers, and 56.5% (2223/3937) had a secondary education. Only 5.2% (203/3937) of schoolchildren’s homes did not have internet access; such access was through WiFi (2151/3937, 54.6%) and mobile internet (1314/3937, 33.4%). In addition, 95.3% (3753/3937) of schoolchildren’s homes had a mobile phone; 31.3% (1233/3937) had computers. In relation to children’s knowledge on healthy eating, 42.2% (1663/3937) of schoolchildren did not know the recommendation to consume at least 5 servings of fruits and vegetables daily, 46.7% (1837/3937) of schoolchildren did not identify front-of-package warning labels (FOPWLs), and 63.9% (2514/3937) did not relate the presence of an FOPWL with dietary risk. Most schoolchildren (3100/3937, 78.7%) preferred to use a mobile phone. Only 38.3% (1509/3937) indicated they preferred a computer. In addition, 47.9% (1885/3937) of caregivers considered that the internet helps in the education of schoolchildren, 82.7% (3254/3937) of caregivers gave permission for schoolchildren to play games with digital devices, and 38% (1495/3937) of caregivers considered that traditional digital games for children are inadequate.

**Conclusions:**

The results suggest that knowledge about nutrition in Peruvian schoolchildren has limitations. Most schoolchildren have access to the internet, with mobile phones being the device type with the greatest availability and preference for use. Caregivers’ perspectives on games and schoolchildren, including a greater interest in using digital games, provide opportunities for the design and development of serious games to improve schoolchildren’s nutritional knowledge in Peru. Future research is needed to explore the potential of serious games that are tailored to the needs and preferences of both schoolchildren and their caregivers in Peru in order to promote healthy eating.

## Introduction

Food intake provides not only energy but also a variety of nutrients that play a crucial role in human health. The diversity and properties of these nutrients continue to be the subject of study to date [1]. Importantly, certain dietary patterns are critical for preventing and addressing the development of chronic noncommunicable diseases, such as cardiovascular disease, cancer, stroke, and diabetes [2] , as well as contributing significantly to the obesity and overweight epidemic [3]. Risks associated with diet, such as low intake of fruits, vegetables, and whole grains, as well as excessive consumption of red meat, processed meats, and sugar-sweetened beverages, are major contributors to global mortality rates [4]. In contrast, a healthy diet not only promotes general well-being but also plays an essential role in the prevention of the aforementioned diseases [5]. Despite the widely recognized benefits of healthy eating for children's health and optimal development, it is concerning to note that many do not meet the established recommendations for fruit and vegetable consumption [6]. Instead, they tend to overconsume sugars and fats, which represents a significant challenge for the promotion of appropriate eating habits in this population [7].

The COVID-19 pandemic accelerated the growing trend of digital media use, especially among children and adolescents [8,9]. Consequently, there has been increasing interest in exploring the use of digital resources to address the global epidemic of overweight, as different studies have reported positive effects on weight reduction in children and adolescents especially in higher-income countries [10,11]. Indeed, health promotion interventions to prevent obesity that seek to engage children through, for example, playful strategies (eg, games) can take advantage of the predisposition for entertainment and learning so that the player can make choices or decisions through the game challenges [12]. In contrast, traditional interventions (eg, weight control in children's centers) may not be appropriate for certain populations with limited mobility, time, or money, especially in low- and middle-income countries (LMICs) [10].

One type of intervention for children using digital media is digital games for educational purposes, also called serious games, which seek to entertain while supporting serious purposes such as education, training, and improving health [13]. Compared with traditional digital games, which mostly prioritize fun and entertainment, health-related serious games are intentionally created for learning about topics such as nutrition or to support health prevention and rehabilitation, for example, so that the participant can receive motivation aids and subsequently achieve a healthy target behavior [14]. In the context of changing eating-related behaviors, some systematic reviews have shown that most studies using serious games had positive results and are suitable to accompany strategies for the prevention and treatment of childhood overweight [15,16]. In recent years, serious games for health promotion, in particular for healthy eating [17], have been shown to be an appropriate alternative for an audience that is increasingly indifferent to television or printed advertisements; even the cost is comparatively lower [18].

Currently, there are several models for the development of digital game interventions; these models offer guidance in each part of the development cycle, from the exploration of the user's needs to the implementation of the intervention. Several researchers have pointed out that one of the main barriers to the development of digital health interventions in LMICs remains the lack of evidence regarding contextual issues, such as specific socioeconomic and infrastructural factors, as well as the target population’s use of and preferences for digital devices and media [19-22]. Inclusion of the target audience is recommended for the development of effective interventions [23]. In that sense, it is important to explore certain characteristics of children and adolescents to identify specific requirements of their context, as well as to establish the learning objectives of the intervention [24]. For this reason, it is necessary to conduct formative research to engage with participants to better understand their needs and the personal relevance of the messages and activities contributing to better-informed serious game interventions [14].

Formative research is a necessary step before developing an intervention because it allows understanding of the complexity of implementation projects, analyzing aspects of responses to change, adaptations, and context [25]. However, not all interventions are developed under a step-by-step implementation scheme. Although effectiveness trials or impact evaluations are required, it is also necessary to publish formative research that serves as a basis to gather design requirements for the development of an intervention [24]. This is especially true for LMICs such as Peru, where there is less evidence on the context and problems of schoolchildren, which makes it difficult to transform pilot programs into sustainable and scalable interventions [26,27].

Given the potential use of game-based approaches with Peruvian schoolchildren to develop health promotion interventions and prevent overweight and obesity, this formative research aimed to explore primary schoolchildren’s knowledge about healthy eating, as well as access to, use patterns of, and preferences for digital media. This information will elucidate design elements and considerations to inform the future development of health interventions such as serious games to promote healthy eating in schoolchildren in the Peruvian context.

## Methods

### Design

In this cross-sectional, exploratory study, information was collected through online surveys for primary schoolchildren and only one of their caregivers. The child’s father, mother, or other relative in charge of the schoolchild at home was considered as the “caregiver.” Based on the last census, public schools in metropolitan Lima located in districts with higher population density were invited to participate.

### Population and Sample

In coordination with the “Regional Directorate of Education of Metropolitan Lima” (RDEML), schoolchildren and their caregivers from the 4th, 5th, and 6th grades of primary school from 17 public schools were invited to participate. A total of 6396 officially registered schoolchildren were invited in 2021. The invitation was sent through WhatsApp groups in which teachers shared the link to the digital questionnaire and a 2-minute informative video to the caregivers. The video presented the study and showed key guidelines for the correct completion of the questionnaire. The data collection took place during October 2021 and November 2021.

### Research Context

Data collection occurred during the 2021 school year, during which classes were entirely remote, and following COVID-19 public health restrictions. Communication between teachers and caregivers of schoolchildren took place using online means through WhatsApp groups or emails. Metropolitan Lima is the capital of the country with approximately 11 million citizens, representing 30% of the national population [28]. Despite the economic development of the country in recent years, even in urban areas, there is a large portion of the population with social vulnerability and scarce economic resources; this population generally uses public schools for the education of their children. The food environment of schoolchildren is disproportionately composed of the availability of ultraprocessed foods, rather than healthy options [29].

### Questionnaire: Development and Design

The first version of the questionnaire was elaborated by the researchers, trying to incorporate the necessary questions for the study variables. The study variables were informed by a literature review. Subsequently, the questionnaire underwent content validation by 8 Peruvian experts from public and private academic institutions with expertise in nutrition, psychology, teaching, engineering, and digital game development. For the content evaluation, the Aiken V coefficient was calculated for each section of the questionnaire, and values > 0.7 were obtained, indicating adequate consensus of the experts [30]. Likewise, modifications were made to the questionnaire in view of the suggestions made by the experts and considered pertinent by the research team. For the form validation, 3 mothers and their children were interviewed through synchronous video calls via Google Meet; in this phase, the caregivers and children were asked if the questions were understandable and if the categories corresponded adequately to their answers. Based on the feedback from these stages, modifications were made to the questionnaire. Subsequently, the questionnaire was digitized using the SurveyMonkey web platform, and informed consent was added at the beginning of the digital questionnaire. Finally, a pilot was carried out with schoolchildren from 1 school that was not part of the 17 schools selected for the research; the link was sent to a group of 30 schoolchildren to complete the questionnaire, in order to validate the process of entering and storing information and to calculate the time it takes to answer the questionnaire (average: 20 minutes; minimum time: 16 minutes). These responses were excluded from the final analysis.

The questionnaire was designed to be answered by schoolchildren and their main caregiver at the same time. It was divided into questions for the child and questions for the caregiver. The introduction to the 2 sections presented the informed consent for the caregiver and the informed assent for the schoolchild, which included information about the research, voluntary participation, and data protection. The first part of the survey was for the schoolchild, with questions on nutritional knowledge (21 items), digital media preferences (21 items), and serious games (24 items). Most questions for schoolchildren included reference images to improve their understanding. The second part of the questionnaire was intended for the caregiver, with questions about eating habits at home related to the schoolchild (36 items) and perceptions of schoolchildren's use of digital media (12 items). All questions were close ended with multiple or single response options. The questions were short and easy to understand. The questions on nutritional knowledge included the option “I do not know” to prevent incorrect answers. The information gathered through these questions was used to construct the indicators of nutritional knowledge presented in the results (its construction is detailed in Multimedia Appendix 1).

### Statistical Analysis

**Figure 1:**
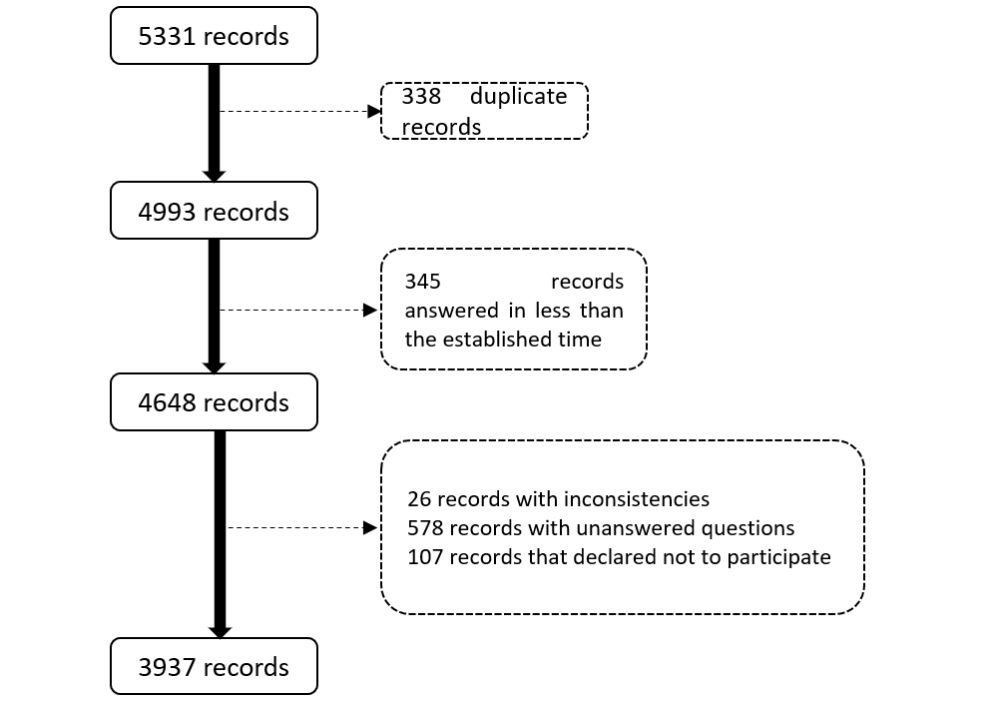
Flowchart of the quality control of the records prior to descriptive analysis.

Initially, 5331 records were downloaded (survey responses submitted to the Survey Monkey platform). Subsequently, quality control was performed on the downloaded database using a systematic process [31]. We excluded 338 duplicate questionnaires from the analysis; we identified them through the similarity of the names of the children and caregivers provided during the informed consent. After this procedure, the data were anonymised. Subsequently, 345 questionnaires were excluded because they were completed in less than the minimum time estimated in the pilot (16 minutes). In addition, 26 questionnaires with inconsistencies and 578 questionnaires with unanswered questions were discarded. The responses of the pilot participants were also excluded from the analysis. In addition, 107 questionnaires without caregiver informed consent or informed assent for the schoolchild were excluded. At the end, 3937 questionnaires were included in the analysis (Figure 1).

Descriptive analysis was performed focusing on variables related to the development of serious play: knowledge about nutrition, caregivers' perceptions about the use of schoolchildren's digital media, and schoolchildren's preferences for a digital intervention. Frequencies and percentages were calculated using Microsoft Excel 365 (Microsoft Corp) and SPSS version 25 (IBM Corp).

### Ethics Approval

The research protocol was approved by the Institutional Research Ethics Committee of the National Institute of Health (Code OI-002-21). We coordinated with the RDEML to implement the research in all the selected schools. The digital questionnaire included informed consent for the caregiver and informed assent for the child. Participation was completely voluntary and could be stopped at any time; there was no compensation.

## Results

Table 1 shows an overview of the characteristics of our participants. The schoolchildren had a mean age of 10.8 years; 51.6% (2030/3937) were female. Most of the caregivers who responded to the survey were the schoolchildren's mothers (3267/3937, 83%) with a mean age of 38.4 years; 56.5% (2223/3937) of the schoolchildren's caregivers had a high school education. During the study period, most of the households had mobile phones (3753/3937, 95.3%) and their own WiFi internet connection (2151/3937, 54.6%).

**Table 1:** Characteristics of schoolchildren in 17 public schools in metropolitan Lima, Peru, 2021 (n=3937).

Variable			Results	
Age of schoolchild (years), mean (SD)			10.8 (1.03)	
Age of schoolchild (years), range			8-15	
**Age of schoolchild (years), n (%)**				
	8-10	1647 (41.8)		
	11-12	2198 (55.8)		
	≥13	92 (2.3)		
**Sex of schoolchild, n (%)**				
	Male	1907 (48.4)		
	Female	2030 (51.6)		
**Role of the surveyed caregiver, n (%)**				
	Father	474 (12)		
	Mother	3267 (83)		
	Relative (uncles, grandparents, siblings)	181 (4.6)		
	No immediate family	15 (0.4)		
Age of surveyed caregiver (years), mean (SD)			38.4 (7.53)	
Age of surveyed caregiver (years), range			18-78	
**Age of surveyed caregiver (years), n (%)**				
	18-30	577 (14.7)		
	31-50	3113 (79.1)		
	≥51	247 (6.3)		
**Mother's educational level^a^, n (%)**				
	No education	44 (1.1)		
	Initial	12 (0.3)		
	Primary	651 (16.5)		
	Secondary	2223 (56.5)		
	Higher education (university or institute)	939 (23.9)		
	Do not know	24 (0.6)		
	The child does not live with the mother	44 (1.1)		
**Devices available in the home^b^, n (%)**				
	Computer	1233 (31.3)		
	Laptop	793 (20.1)		
	Mobile phone	3753 (95.3)		
	Tablet	635 (16.1)		
**Type of internet access^b^, n (%)**				
	WiFi inside the home	2151 (54.6)		
	Family or neighbor's WiFi	603 (15.3)		
	Mobile internet (mobile phone)	1314 (33.4)		
	No internet access at home	203 (5.2)		

^a^The question referred exclusively to the schoolchild’s mother.

^b^The variables were collected independently for each category, and the participant could choose more than one option.

In Table 2, we present an overview of schoolchildren’s knowledge about healthy eating from our study: 90.8% (3574/3937) of the schoolchildren were able to distinguish, on 3 occasions, the best food options for a healthy lunchbox, and 42.2% (1663/3937) did not know the recommendation to consume at least 5 servings of fruits and vegetables per day. With respect to the questions asking schoolchildren to identify the foods with the highest sugar and fat content, 18.3% (720/3937) of the participants could not identify the solid food with the highest sugar content, 10.8% (426/3937) did not identify the beverage with the highest sugar content, and 8% (316/3937) did not identify the food with the highest fat content. In addition, 53.3% (2100/3937) of schoolchildren did not identify the front-of-package warning labels (FOPWLs) on the food images displayed. Furthermore, 36.1% (1423/3937) of schoolchildren did not relate the presence of FOPWL with the consequences they would experience if they consumed these products in excess. Finally, 95.5% (3755/3937) of schoolchildren indicated that their parents are the ones who teach them about healthy eating. All questions are shown in Multimedia Appendix 1.

**Table 2:** Knowledge about healthy eating among schoolchildren in 17 public schools in metropolitan Lima, Peru, 2021 (n=3937).

Variable		Boys, n (%)	Girls, n (%)	All, n (%)
**Assembled a healthy lunch box^a^**				
	Not achieved	167 (8.8)	196 (9.7)	363 (9.2)
	Achieved	1740 (91.2)	1834 (90.3)	3574 (90.8)
**Knows the recommendation of at least 5 servings of fruits and vegetables a day**				
	Met	1098 (57.6)	1176 (57.9)	2274 (57.8)
	Not known	809 (42.4)	854 (42.1)	1663 (42.2)
**Identifies the solid food with the highest sugar content^b^**				
	Not identified	389 (20.4)	331 (16.3)	720 (18.3)
	Identified	1518 (79.6)	1699 (83.7)	3217 (81.7)
**Identifies the beverage with the highest sugar content^c^**				
	Not identified	237 (12.4)	189 (9.3)	426 (10.8)
	Identified	1670 (87.6)	1841 (90.7)	3511 (89.2)
**Identifies the food with the highest fat content^d^**				
	Not identified	152 (8)	164 (8.1)	316 (8)
	Identified	1755 (92)	1866 (91.9)	3621 (92)
**Identifies the front-of-package warning label (FOPWL) on food packaging^e^**				
	Not identified	1024 (53.7)	1076 (53)	2100 (53.3)
	Identified	883 (46.3)	954 (47)	1837 (46.7)
**Relates unhealthy foods to the presence of FOPWL^f^**				
	No	710 (37.2)	713 (35.1)	1423 (36.1)
	Yes	1197 (62.8)	1317 (64.9)	2514 (63.9)
**Provider of food information^g,h^**				
	My teachers	608 (31.9)	693 (34.1)	1301 (33)
	My parents	1813 (95.1)	1942 (95.7)	3755 (95.4)
	My friends	32 (1.7)	50 (2.5)	82 (2.1)
	Health personnel	350 (18.4)	363 (17.9)	713 (18.1)
	I do not remember	19 (1)	16 (0.8)	35 (0.9)

^a^“Achieved”: the schoolchild chose 3 appropriate options: cookie versus apple, home-produced traditional drink versus soda, chicken sandwich versus chocolate cake.

^b^“Identified”: the schoolchild chose the chocolate cake instead of the banana or cookies.

^c^“Identified”: the schoolchild chose the glass of soda instead of the glass of water or lemonade.

^d^“Identified”: the schoolchild chose potato chips with sauce instead of boiled potato or potato chips alone.

^e^“Identified”: the schoolchild selected foods with a nutritional FOPWL twice.

^f“^Yes“: the schoolchild associated the most harmful food with the one that had an FOPWL twice.

^g^Who teaches you to eat healthy?

^h^The categories were collected independently for each category (Yes/No).

In Table 3, we present an overview of caregivers’ perceptions about their children’s use of digital media. Parents reported that the most used device by schoolchildren to distract themselves was a mobile phone (2514/3937, 63.9%); likewise, the websites with the highest use were YouTube (1178/3937, 29.9%) and TikTok (723/3937, 18.4%). Regarding the perception of internet use in the education of their children, most caregivers (1885/3937, 47.9%) maintained a cautious position by selecting the option ”It helps, but it can be harmful.“ Regarding attitudes toward the use of devices for entertainment, very few caregivers indicated that they would not give permission for any reason (243/3937, 6.2%). Regarding parental control, many caregivers said that a time limit can be set (2611/3937, 66.3%). Finally, many caregivers (1495/3937, 38%) considered that the use of digital games may be inappropriate for their children.

**Table 3:** Perceptions of caregivers about schoolchildren's digital media use in metropolitan Lima, Peru, 2021 (n=3937).

Variable			Caregivers of boys, n (%)		Caregivers of girls, n (%)		All caregivers, n (%)
**Children's most preferred device for distraction^a^**							
	Computer	237 (12.4)		203 (10)		440 (11.2)	
	Laptop	98 (5.1)		103 (5.1)		201 (5.1)	
	Mobile phone	1188 (62.3)		1326 (65.3)		2514 (63.9)	
	Tablet	162 (8.5)		183 (9)		345 (8.8)	
	Nintendo, Playstation	58 (3.0)		10 (0.5)		68 (1.7)	
	Other (TV, Gameboy)	164 (8.6)		205 (10.1)		369 (9.4)	
**Your children's favorite digital media for distraction^b^**							
	Facebook, Instagram	39 (2)		57 (2.8)		96 (2.4)	
	YouTube	586 (30.7)		592 (29.2)		1178 (29.9)	
	TikTok	143 (7.5)		580 (28.6)		723 (18.4)	
	WhatsApp	157 (8.2)		194 (9.6)		351 (8.9)	
	Mobile games	677 (35.5)		360 (17.7)		1037 (26.3)	
	Computer games	141 (7.4)		82 (4)		223 (5.7)	
	Google	76 (4)		90 (4.4)		166 (4.2)	
	I do not know	88 (4.6)		75 (3.7)		163 (4.1)	
**Perception of the internet in their children's education^c^**							
	Helps a lot	552 (28.9)		579 (28.5)		1131 (28.7)	
	Sometimes it helps	389 (20.4)		461 (22.7)		850 (21.6)	
	Helps, but can be harmful	934 (49)		951 (46.8)		1885 (47.9)	
	Does not help	20 (1)		25 (1.2)		45 (1.1)	
	No clear opinion	12 (0.6)		14 (0.7)		26 (0.7)	
**Attitude toward the use of digital devices for entertainment^d^**							
	I always give him/her permission	94 (4.9)		86 (4.2)		180 (4.6)	
	I hardly notice when he/she plays	133 (7)		127 (6.3)		260 (6.6)	
	I can give him/her permission, it depends on	1566 (82.1)		1688 (83.2)		3254 (82.7)	
	I do not give him/her permission	114 (6)		129 (6.4)		243 (6.2)	
**Parental control of the use of digital devices for entertainment purposes^e^**							
	Yes, he/she always obeys me	1235 (64.8)		1376 (67.8)		2611 (66.3)	
	Regular	484 (25.4)		493 (24.3)		977 (24.8)	
	Difficult	69 (3.6)		59 (2.9)		128 (3.3)	
	No, it is very difficult	119 (6.2)		102 (5)		221 (5.6)	
**Perception of the use of digital games for schoolchildren^f^**							
	Very adequate	37 (1.9)		56 (2.8)		93 (2.4)	
	Suitable	265 (13.9)		292 (14.4)		557 (14.1)	
	I have no problem using it	553 (29)		585 (28.8)		1138 (28.9)	
	Inadequate	737 (38.6)		758 (37.3)		1495 (38)	
	Very inadequate	315 (16.5)		339 (16.7)		654 (16.6)	

^a^According to your perception, which device does your child prefer to be distracted by?

^b^When your child is out of school hours and connects to the internet, what type of site does he/she visit or prefer the most?

^c^At present, what is your perception of the internet in your child’s education?

^d^When your child wants to use the devices for entertainment, what is your attitude?

^e^When your child is entertained using devices, can you set a time limit?

^f^What is your perception of digital games used by children?

In Table 4, we present the schoolchildren’s source of distraction and preferences in relation to digital games. Most schoolchildren were distracted by mobile phone use (3100/3937, 78.7%). Schoolchildren reported using digital games when they are bored (2211/3937, 56.2%) or when they feel like it (1308/3937, 33.2%). Most schoolgirls were interested in using a serious game that teaches them about food (3280/3937, 83.8%). Regarding the digital game character, 31.8% (636/2030) of girls preferred animated animals, while 42.4% (798/1907) of boys preferred superheroes. Regarding the environment of the digital game, many schoolchildren (1715/3937, 44%) preferred a nature environment.

**Table 4:** Preferences for digital games of schoolchildren in 17 public schools in metropolitan Lima, Peru, 2021 (n=3937).

Variable			Boys, n (%)		Girls, n (%)		All, n (%)
They are distracted by using PCs.^a^			768 (40.3)		741 (36.5)		1509 (38.3)
They get distracted by using mobile phones.^b^			1494 (78.3)		1606 (79.1)		3100 (78.7)
They are distracted by using a tablet.^c^			434 (22.8)		429 (21.1)		863 (21.9)
**Times that digital games are used^d,e^**							
	When I feel like it	644 (33.8)		664 (32.7)		1308 (33.2)	
	When I am happy	162 (8.5)		182 (9)		344 (8.7)	
	When I am sad	84 (4.4)		105 (5.2)		189 (4.8)	
	When I'm bored	1073 (56.3)		1138 (56.1)		2211 (56.2)	
	When I am with friends	395 (20.7)		289 (14.2)		684 (17.4)	
	When I am alone at home	364 (19.1)		389 (19.2)		753 (19.1)	
	I do not play	170 (8.9)		311 (15.3)		481 (12.2)	
	I do not know	59 (3.1)		54 (2.7)		113 (2.9)	
**Interest in a digital nutritional game^f^**							
	I am interested	1559 (82.3)		1721 (85.3)		3280 (83.8)	
	I have little interest	218 (11.5)		180 (8.9)		398 (10.2)	
	I do not know	74 (3.9)		65 (3.2)		139 (3.6)	
	I am not interested	44 (2.3)		52 (2.6)		96 (2.5)	
**Preferences for digital game characters^g^**							
	Animated animals	503 (26.7)		636 (31.8)		1139 (29.3)	
	Superheroes	798 (42.4)		426 (21.3)		1224 (31.5)	
	Fantastic animals	124 (6.6)		191 (9.5)		315 (8.1)	
	Children	214 (11.4)		309 (15.4)		523 (13.5)	
	Teenagers	245 (13)		440 (22)		685 (17.6)	
**Digital game environment preference^h^**							
	Magical	422 (22.4)		664 (33)		1086 (27.9)	
	Nature	803 (42.5)		912 (45.4)		1715 (44)	
	Universe	353 (18.7)		108 (5.4)		461 (11.8)	
	City	310 (16.4)		326 (16.2)		636 (16.3)	

^a^When you want to be distracted, do you use a computer?

^b^When you want to be distracted, do you use your mobile phone?

^c^When you want to be distracted, do you use a tablet?

^d^At what time do you play on the computer, mobile phone, or tablet?

^e^The categories were collected independently for each category, and the participant could choose more than one option.

^f^Would you like to try a digital game that teaches you how to eat well like in the previous figure?

^g^What kind of character would you like to have in a digital game?

^h^What kind of environment would you like a digital game to have?

## Discussion

### Nutritional Knowledge of Schoolchildren: Challenges to Promoting Healthy Eating

The results suggest that primary schoolchildren have limited knowledge about nutrition. Although most of the schoolchildren from our study knew how to put together a healthy lunch box and identified high-calorie foods, only 42.5% of them knew the recommendation to consume at least 5 portions of fruits and vegetables per day. The “5 a day” message is part of an international campaign recommended by the World Health Organization (WHO) for healthy eating [32]. Our results show that, although this recommendation is shared by public and private institutions in Peru, through health promotion campaigns, information leaflets at schools, and TV advertisements, this message has not yet improved the knowledge of schoolchildren. Our results are very similar to an educational intervention study with Chilean children in which González et al [33] showed that, at baseline in 2018, only 45.6% of the children were aware of this message [33].

Another aspect evaluated was related to the healthy eating policy, which was implemented in Peru in 2019 [34] and requires that processed foods have FOPWLs. Our study shows that 53.3% of schoolchildren did not identify the FOPWL in the 2 images of ultraprocessed foods presented to them. The FOPWL system is a type of front-of-package (FOP) labeling that aims to make product nutritional information more understandable to consumers [35] and encourage healthier food choices [36]. The fact that schoolchildren do not recognize FOPWL means that they have not received sufficient information about nutrition labeling in their schools nor in their homes even though they are able to identify them. It has been shown that children aged 7 years to 13 years understand nutrition information and can use it to classify healthy and unhealthy foods [37,38]. FOPWL focuses on helping consumers make better-informed food-related choices with the aim of potentially discouraging the purchasing and consumption of ultraprocessed foods by highlighting the unhealthy aspects of products by pointing out their health risks (eg, ”High in saturated fats“). According to our results, 36.1% of schoolchildren participants did not associate the presence of FOPWL with unhealthy foods. These results are similar to those of a study with Brazilian schoolchildren in which the FOPWL was the most accepted type of FOP, compared with the Guideline Daily Amount (GDA) label and the nutritional traffic light, but it did not play the expected dissuasive role [39]. Reducing the intake of ultraprocessed foods is a key factor in the prevention of excess weight and various metabolic diseases [40,41]. Different studies have indicated that ultraprocessed foods, compared with natural or minimally processed foods, are calorie dense; have high concentrations of free sugars, sodium, and saturated fats; and have low concentrations of fiber and micronutrients [42,43].

In this study, the majority of schoolchildren (95%) indicated that their parents teach them about healthy eating. Children's eating habits are closely related to behaviors at home, specifically to the parenting and eating styles of the parents [44]. For this reason, interventions that can be targeted at parents or involving them could offer many opportunities in enhancing healthy eating practices by children, such as increasing the consumption of fruits and vegetables [45].

### Opportunities for Serious Games: Digital Game Preferences of Schoolchildren

Almost all schoolchildren (83.8%) affirmed their interest in trying a digital game that teaches them about healthy eating. This result relates to the wide acceptability of digital games for children and adolescents, which could influence the use of serious games for health and nutrition. The acceptability of games by children is key to developing effective and better-informed interventions, as the enjoyment and participation of children increase the chances of achieving a change in eating behavior [46]. Most schoolchildren in our study preferred to play when they are bored (56.2%) or when they are in the mood to play (33.2%); these results are similar to those in the study by Holzmann et al [47] in which German children and adolescents had a positive emotionally induced digital game experience for pleasure and boredom [47]. Thus, the fact that most schoolchildren use digital games when they are in a good mood could be exploited to suport active learning in an entertaining way through serious games. Several authors have studied the use of serious games to improve children's nutritional knowledge [48,49]. In order to transmit knowledge using a game, several elements must be considered in the design, such as the character or the environment. In that sense, our study showed that most girls preferred animated animals (31.8%), while boys had a greater affinity for superheroes (42.4%). In relation to the environment of the digital game, the greatest preference of schoolchildren was a context with nature (44%). Formative studies prior to game development have investigated these preferences and had similar findings; for example, in the study by Holzmann et al [47], the majority of schoolgirls preferred a heroic animated human character, and in the study by Kayali et al [50], the majority of Austrian children aged 8 years to 14 years preferred animals and a natural environment for the digital game [50]. This information is essential because children's preferences for characters influences their motivation to use serious games that can support learning about nutrition, as children remember nutritional information when they are presented with a sympathetic character [51].

### The Role of Caregivers in the Development of Serious Games

Taking a human-centered approach [52], our first step was to include caregivers in the survey because of their clear relevance in the education of schoolchildren [53]. We inquired about caregivers’ perceptions and attitudes regarding the use of the internet and digital games by schoolchildren. Considering that, during the COVID-19 pandemic, the dependence on digital media at home increased [54], and this may have affected how parents mediated the use of this technology with their children. For example, caregivers usually share mobile phones with their children; therefore, they can give an informed opinion about certain behaviors of schoolchildren. Most caregivers (47.9%) from our study considered that the internet helps in the education of schoolchildren, but they were aware that it can be harmful. The major concerns include risks such as cyberbullying or inappropriate content [55]. This concern often causes caregivers to apply restrictive measures such as setting rules for digital media use for their children. In our study, the majority of caregivers (82.7%) indicated that they permitted the use of digital devices by their children for entertainment. In addition, the majority of caregivers noted that schoolchildren complied with the time limits they agreed to for the use of digital devices for entertainment (66.3%). Parental mediation aims for children to achieve self-regulation and digital skills that allow the child to limit the risks related to the use of digital media and thus maximize the benefits digital media offers [56]. Therefore, the acceptability of a novel digital intervention for caregivers may be contingent on the serious game and careful consideration of the aforementioned risks.

In relation to digital games, 38% of caregivers perceived that the use of digital games for schoolchildren is inadequate. These results are similar to those of a study of schoolchildren and parents in New Zealand [57], in which parents were involved in the development of a serious game for nutrition education. A major concern was the excessive screen time caused by digital games, which increased sedentary behavior. Recommendations such as the Canadian 24-hour Movement Guidelines state that children have less than 2 hours per day of screen time after school [58]. Thus, more research is needed to understand whether a digital game for health, such as a serious game, can be incorporated into recreational or educational screen time, especially in LMICs. In addition, our results could be interpreted to mean that parents do not tend to give a positive rating to leisure time activities for 8- to 12-year-olds, compared with early childhood [59]. Parents tended to pigeonhole the use of digital games into totally playful and interactive purposes, as serious games are not popular in Peruvian society. Future research shall explore the potential value of serious games to support and improve children’s health in the Peruvian context. If parents are involved and become aware of the potential opportunities that serious games for health could offer [60] , their perceptions may change.

### Implications for Serious Game Design in the Peruvian Context

The results of the study indicate that the best device to deploy a serious game would be a mobile phone, since it is the device type most accessible in households (95.5%) and the most used for distraction according to the schoolchildren themselves (78.7%) and their caregivers (63.9%). Due to the growing popularity of mobile phones and apps, several health apps aimed at modifiable risk factors such as children's diet have been developed [61]. Interventions based on the use of mobile phones are often effective at improving behavioral changes associated with obesity in children aged 8 years to 12 years [62].

Likewise, our study highlights how feasible it could be to develop an online game in urban areas of Peru, since almost all participant households had internet at home (94.8%) and most participant households were connected to the internet through WiFi (69.9%), which generally has unlimited megabytes. These results are similar to those of the Residential Survey of Telecommunications Services [63], which indicated that 95% of households in metropolitan Lima have internet access and the main source of internet access was through WiFi (68.5%) and mobile phones (92.3%). The residential survey also reported that 94.6% of households have a smartphone [63].

Our study suggests that serious games may be used as an educational tool to increase schoolchildren's knowledge about food and nutrition. To date, there have been no large-scale interventions in Peru to improve the nutritional knowledge of schoolchildren. In addition, educational content on nutrition in public schools is almost nonexistent, as it competes with other activities in the school curriculum.

Serious games are more cost-effective for reaching large numbers of participants than traditional interventions, such those using human resources; LMICs generally have limited resources for training and transportation of intervention staff [64]. In addition, serious games may not disrupt classroom activities and may even fit harmoniously into school curricula, such as the “Fitter Critters” serious game that children play in health classrooms for 1 week [65]. Finally, serious games can offer flexibility in relation to the location or time of play without negatively affecting the content of the intervention. Future research should further engage with caregivers and schoolchildren to explore the design, feasibility, and acceptability of the games in the Peurivan context.

### Importance of Developing Digital Intervention to Improve Schoolchildren's Nutrition

Today's food environment is characterized by increased availability of cheap, tasty, energy-dense foods, coupled with wide-ranging and highly persuasive food marketing [66]. Most foods intended for children are generally excessive in sugars fat, and sodium [67,68]. In that sense, children and adolescents constitute a vulnerable group that deserves social protection, as they have limited nutritional knowledge, they are unable to perceive the risks of their behaviors, and their choices may be affected by the sociocultural environment such as the marketing of unhealthy foods.

Vulnerability also refers to the socioeconomic level of the students in this study; although a specific indicator was not evaluated to determine socioeconomic level, it is very likely that most students belonged to a low socioeconomic stratum. Students in public schools are generally of low socioeconomic status; this is because private schools in Peru enjoy better prestige and involve high expenses for families. In this sense, socioeconomic status is one of the main determinants of food choice and eating habits; households with lower incomes buy less healthy food [69,70]. For example, adolescents of low socioeconomic status often choose foods for their snacks with higher sugar content compared with their peers of higher socioeconomic status [71]. It should be noted that most successful experiences at improving nutrition in schoolchildren have been reported mainly in contexts other than LMIC [72]. It is essential that interventions such as serious games are aligned to promote children's health and development in a digital world, establishing responsible, conscious use of the screen as well as ensuring the participation of caregivers [73].

### Strengths and Limitations

To our knowledge, our study is the first formative research carried out with primary schoolchildren in metropolitan Lima to support future development of a digital intervention to improve nutrition. Despite Peru's economic progress in recent years, the insufficient decentralization of the country has meant that metropolitan Lima represents 30% of the country's total population. For this reason, an attempt was made to cover the largest number of participants in the study (3937 schoolchildren), and schools in the most densely populated districts were included. In addition, the questionnaire was developed by a multidisciplinary team with expertise in health sciences, education, and informatics, disciplines relevant to the type of intervention to be developed. To achieve the best quality of responses, interviews and a pilot were conducted, the results of which served to not only improve the understanding and accuracy of the questions but also establish criteria for quality control of the database.

There are some limitations that should be mentioned. The results may be prone to selection bias, since by using a digital survey, participation may have been limited to those with internet access. In addition, probability sampling was not used, so the results are not representative of metropolitan Lima. Although the questions on nutritional knowledge took into account aspects of the food guide for the Peruvian population [74], these aspects are not yet considered in the official curriculum. There is no official school curriculum on nutrition in Peru. Finally, considering that a self-administered survey with many questions could fatigue participants and lead to decreased answer quality, we decided to prioritize the most relevant questions to answer the purpose of the research. This led to disregarding questions to better characterize the population, such as those related to socioeconomic status, and we did not include open-ended questions. We believe that this type of information would be better addressed in future research with qualitative methodology. Another limitation of this study lies in the use of self-administered questionnaires, which relied on self-reporting by the schoolchildren and their caregivers.

### Conclusions

The results presented indicate that there is limited knowledge about nutrition by schoolchildren, specifically in the consumption of healthy food (fruits and vegetables) and the management of information on nutrition labels of ultraprocessed products. Moreover, the results revealed that schoolchildren are interested in using serious games to improve nutrition education. Although it seems feasible to develop an intervention using the internet, since most households have access to this service, our results higlights that mobile phones might be the most suitable device to develop a digital intervention, since they are the most available and preferred device by schoolchildren. Another aspect to consider for the development of a serious game intervention is to get the acceptance and trust of the caregivers, both in the type of content and time of use of the serious game. Therefore, the development and implementation of a serious game is a feasible alternative for schools to increase nutrition knowledge and promote healthy eating in schoolchildren as long as all the different stakeholers are involved in the design process of the intervention.

## Appendix

Multimedia Appendix 1 Indicators of nutritional knowledge.

## Abbreviations

GDA: Guideline Daily Amount
FOP: front-of-package
FOPWL: front-of-package warning label
LMIC: low- and middle-income countries
RDEML: Regional Directorate of Education of Metropolitan Lima
WHO: World Health Organization
