# Supplementary material for: Exploring Children's Knowledge of Healthy Eating, Digital Media Use, and Caregivers’ Perspectives to Inform Design and Contextual Considerations for Game-Based Interventions in Schools for Low-Income Families in Lima, Peru: Survey Study
Source: JMIR Form Res. 2024 May 14;8:e49168. doi: 10.2196/49168 (PMC11134240; doi:10.2196/49168)
Supplement: Multimedia Appendix 1 [file formative_v8i1e49168_app1.docx]

**Supplementary material:**

1. **Indicator "Assembled a healthy lunch box":** It was determined that the child "succeeded" in assembling a healthy lunch box if in questions 1.1, 1.2 and 1.3 they marked the apple, natural drink and bread with chicken respectively.
   1. Which of the following foods would you choose for a healthy lunch box?


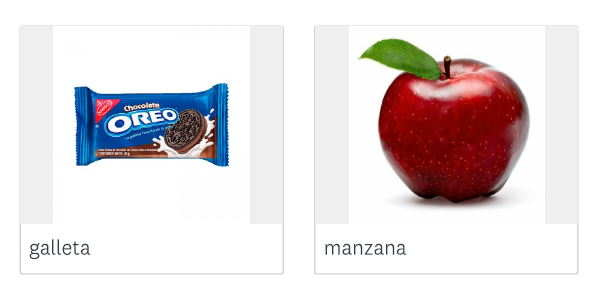


Cookies

Apple

- 1. Which of the following foods would you choose for a healthy lunch box?


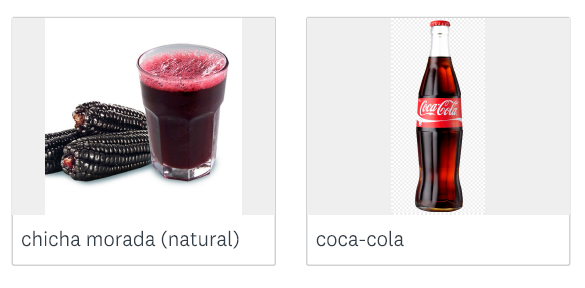


Soda

Home-produced traditional

- 1. Which of the following foods would you choose for a healthy lunch box?


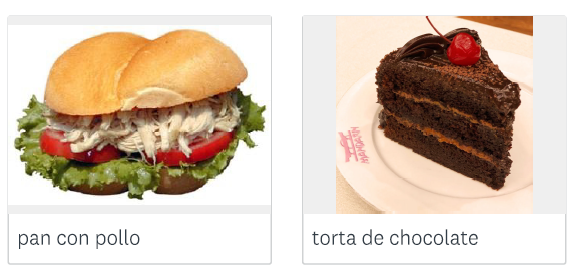


chicken sandwich

chocolate cake

1. **Indicator "Knows the recommendation of at least 5 servings of fruits and vegetables per day":** It was determined that the child **"does not know"** the recommendation if in both questions (2.1 and 2.2) he/she marked option "a" (1 to 2 fruits/vegetables) since adding both options together would not reach the 5 servings of fruits or vegetables recommended daily. It was also determined that the child **"does not know"** if in at least one of the questions he/she checked option "d" (I don't know).
   1. How much fruit should we consume in a day?
2. 1 to 2 fruits
3. 3 to 4 fruits
4. As many as you wish
5. I do not know
   1. How many vegetables should we consume in a day?
6. 1 to 2 vegetables
7. 3 to 4 vegetables
8. As many as you wish
9. I do not know
10. **Indicator "Identifies the solid food with the highest sugar content"**: It was determined that the child "identifies" if in question 3.1 he/she checked the option "chocolate cake"
    1. Of the three foods, which has the most sugar?


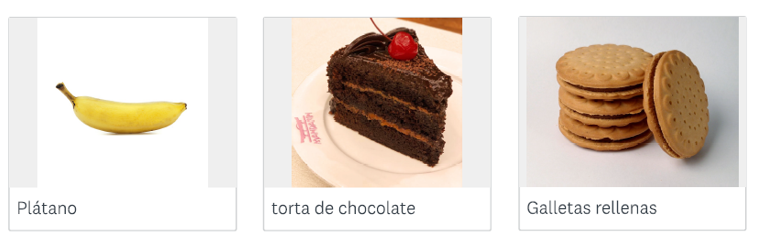


banana

chocolate cake

cookies

1. **Indicator "Identifies the drink with the highest sugar content":** It was determined that the child "identifies" if in question 4.1 he/she checked the option "glass of soda"
   1. Which of the three beverages has the most sugar?


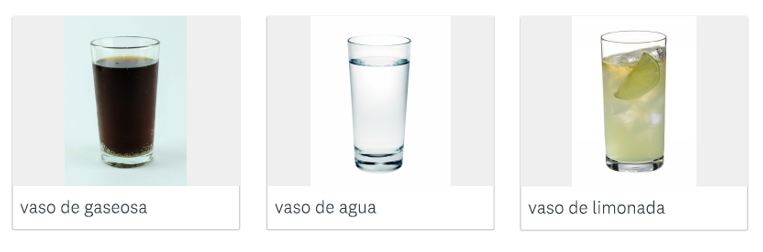


Soda

Water

Limonade

1. **Indicator "Identifies the food with the highest fat content":** It was determined that the child "identifies" if in question 5.1 he/she checked the option "French fries with creams"
   1. Of the three foods, which has the most fat?


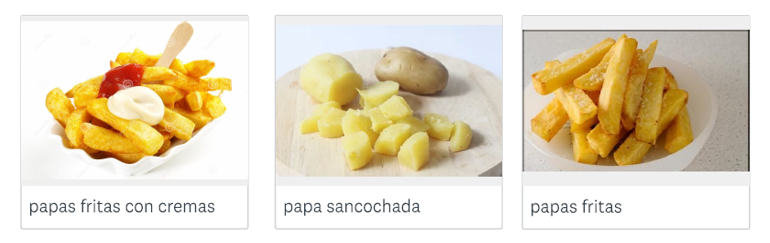


potato chips with sauces

boiled potato

potato chips

1. **Indicator "Identifies FOPWL on food labels":** It is determined that the child "Identifies" if in the 2 questions (6.1 and 6.2) he/she marked the products with FOPWL
   1. Select the cookie that has a FOPWL


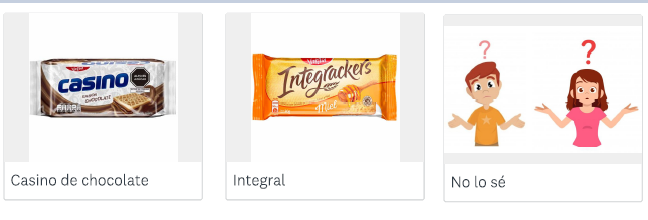


filled biscuit

integral crackers

I don't know

- 1. Select the soda that has a FOPWL


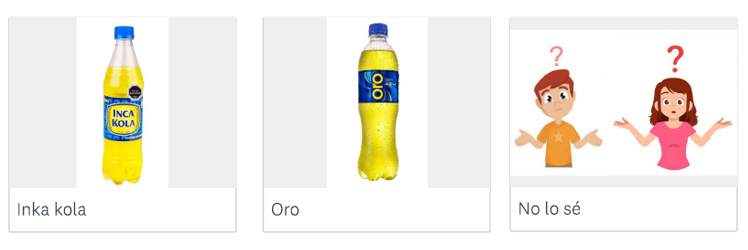


I don't know

Soda option B

Soda option A

1. Indicator "Relates unhealthy foods with the presence of FOPWL ": It is determined that the child "Identifies" if in the 2 questions (6.1 and 6.2) he/she marked the products with FOPWL
   1. What is the most harmful drink?


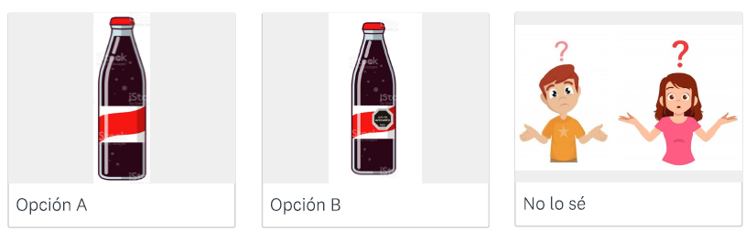


Soda option A

Soda option B

I don't know

- 1. What is the most unhealthy cookie?


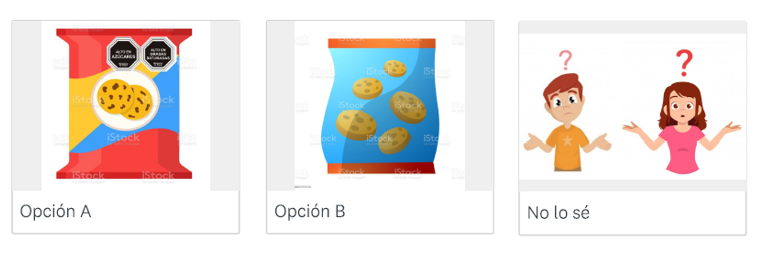


Cookies option A

Cookies option B

I don't know
